# Double Image Encryption System Using a Nonlinear Joint Transform Correlator in the Fourier Domain

**DOI:** 10.3390/s23031641

**Published:** 2023-02-02

**Authors:** Ronal A. Perez, Elisabet Pérez-Cabré, Juan M. Vilardy, María S. Millán, Cesar O. Torres

**Affiliations:** 1 Grupo de Investigación en Física del Estado Sólido (GIFES), Faculties of Basic and Applied Sciences, and Engineering, Universidad de La Guajira, Riohacha 440007, La Guajira, Colombia; 2 Applied Optics and Image Processing Group, Universitat Politècnica de Catalunya · BarcelonaTech, 08222 Terrassa, Barcelona, Spain; 3 Grupo de Óptica e Informática, Department of Physics, Universidad Popular del Cesar, Valledupar 200001, Cesar, Colombia

**Keywords:** double image encryption, joint transform correlator (JTC), Fourier domain

## Abstract

In this work, we present a new nonlinear joint transform correlator (JTC) architecture in the Fourier domain (FD) for the encryption and decryption of two simultaneous images. The main features of the proposed system are its increased level of security, the obtention of a single real-valued encrypted signal that contains the ciphered information of the two primary images and, additionally, a high image quality for the two final decrypted signals. The two images to be encrypted can be either related to each other, or independent signals. The encryption system is based on the double random phase encoding (DRPE), which is implemented by using a nonlinear JTC in the FD. The input plane of the JTC has four non-overlapping data distributions placed side-by-side with no blank spaces between them. The four data distributions are phase-only functions defined by the two images to encrypt and four random phase masks (RPMs). The joint power spectrum (JPS) is produced by the intensity of the Fourier transform (FT) of the input plane of the JTC. One of the main novelties of the proposal consists of the determination of the appropriate two nonlinear operations that modify the JPS distribution with a twofold purpose: to obtain a single real-valued encrypted image with a high level of security and to improve the quality of the decrypted images. The security keys of the encryption system are represented by the four RPMs, which are all necessary for a satisfactory decryption. The decryption system is implemented using a 4*f*-processor where the encrypted image and the security keys given by the four RPMs are introduced in the proper plane of the processor. The double image encryption system based on a nonlinear JTC in the FD increases the security of the system because there is a larger key space, and we can simultaneously validate two independent information signals (original images to encrypt) in comparison to previous similar proposals. The feasibility and performance of the proposed double image encryption and decryption system based on a nonlinear JTC are validated through computational simulations. Finally, we additionally comment on the proposed security system resistance against different attacks based on brute force, plaintext and deep learning.

## 1. Introduction

Many encryption techniques use optical processing systems to take advantage of their ultrafast computational speed, high parallel processing capacity, and also the wide variety of controllable physical parameters. All the mentioned features make optical processors very attractive for information encryption techniques with high levels of security [1,2,3,4,5,6].

The technique of double random phase encoding (DRPE) has been extensively shown as an important technique to encrypt images by using optical means [7,8]. This DRPE has been typically implemented with a joint transform correlator (JTC) architecture [2,4,5,9,10,11,12,13,14]. The JTC architecture for optical encryption systems commonly uses two random phase masks (RPMs) in its input plane with the purpose of converting a single original image into a stationary white noise signal (encrypted image) in the Fourier domain (FD) [5]. The initial JTC-based encryption systems presented in prior works [2,4,9,10,11] were implemented by using linear 2*f* optical processors, which have been shown to be vulnerable against the chosen-plaintext attack (CPA) [15], the known-plaintext attack (KPA) [16,17], the ciphertext-only attack (COA) [18] and an attack based on deep learning [19]. In order to enhance the quality of the decrypted image and to improve the security of the previous linear JTC-based encryption systems, nonlinear JTC architectures were proposed in different optical processing domains [5,6,20,21,22,23,24,25,26,27,28,29]. Up to now, the mentioned nonlinear JTC architectures were applied to encrypt only a single image or piece of information.

In this work, we propose a new extension of the single image encryption system based on a nonlinear JTC [6,20] to a double image encryption system using new nonlinear modifications applied to the JTC architecture in the FD. The purpose of using two images in this paper is to obtain a robust encryption–decryption system for two different pieces of information. Moreover, the simultaneous encryption of the two primary images is achieved in a single step, differently to other works that use a sequential procedure with a number of steps coincident with the number of images to encrypt. The two original images to encrypt are encoded in phase; this fact produces an improvement in the security of the proposed encryption system against CPA, KPA and COA because the phase encoding is a nonlinear operation, and the RPMs bonded to these two original images encoded in phase become security keys [28,30]. All the input plane of the JTC is fully encoded in phase, and it is composed by four non-overlapping data distributions placed side-by-side with no blank spaces between them. These four data distributions are designed by using the two original images encoded in phase and four RPMs. The joint power spectrum (JPS) is the intensity of the Fourier transform (FT) of the input plane of the JTC. The real-valued encrypted image is computed by using two new nonlinear modifications applied on the JPS. We remark that the definitions of these two nonlinear modifications are entirely new and different from the nonlinear modifications presented in previous works of JTC-based encryption systems. The new nonlinear operations introduced in the JPS allow a correct retrieval of the decrypted images, with a remarkable signal quality. In addition to this, the applied nonlinearities break the linear behaviour of previous JTC-based encryption systems that have been shown to be vulnerable to different types of attacks. The security keys of the proposed encryption system are represented by four RPMs with a larger key space.

With respect to other JTC-based systems developed in former works [2,5,6,9,10,11,12,13,14,20,21,22,23,24,25,26,27,28,29], the proposed encryption and decryption system for two images allows the simultaneous encryption of two images with a high level of security for the single real-valued encrypted image, besides retrieving high quality decrypted images, either simultaneously or separately. Our encryption and decryption system is not an iterative algorithm, the definitions of the two nonlinear operations applied on the JPS are entirely new, and the use of a new nonlinear JTC allows for breaking the linearity of former JTC-based encryption systems, aiming to obtain an encrypted image better protected against several attacks.

The paper is organized as follows: Section 2 reviews the state of the art related to the proposal of this work, remarking on the differences and advantages of the current proposal in comparison to other existing systems based on optical processors. In Section 3, the double-image encryption and decryption systems are presented. In Section 4, the simulation results of the proposed system are computed to illustrate the proposal. Finally, Section 5 contains our conclusions.

## 2. Related Works

Several works have been proposed for the encryption and decryption of two images based on optical techniques [31,32,33,34,35,36,37,38,39,40,41,42,43,44,45,46,47,48]. In [31,36], one image to encrypt is encoded in amplitude and the other image is encoded in phase, in order to obtain a single complex-valued input image for the encryption system based on the classical DRPE. The double-image encryption presented in [32] was developed using a linear JTC, two-step-only quadrature phase-shifting digital holography, one image encoded in amplitude and the other image encoded in phase placed at the same location of the input plane of the JTC, in order to generate two encrypted images given by two nonnegative interferograms. Other double image encryption also based on a linear JTC and the two-step phase-shifting digital holography uses two images encoded in amplitude sequentially at the input plane of the encryption system and produces two encrypted images [33]. The double-image encryption in [34] generated two encrypted images by using two images to encrypt encoded in phase and a numerical algorithm based on two channels. A 4*f*-processor is used in [35] to encrypt two images encoded in amplitude into two encrypted images by using an addition and subtraction process in the FD. Other double-image encryption systems are based on wavelength multiplexing [37] and iterative phase retrieval algorithms [38,39,40,41].

In [42,43], the two images to encrypt were encoded in amplitude at different locations at the input plane of a linear JTC architecture. The nonlinear JTC system presented in [44] used several input images, one for encryption and decryption and two more images as keys for authentication of the first one. The last two images were used exclusively for verification purposes of a single input image and not for decryption. The nonlinear operations applied on the JPS in [44] are conceptually different from the nonlinear operations proposed in this paper. The double-image encryption system in [45] was implemented using a nonlinear JTC with one image encoded in amplitude and the other image encoded in phase placed at the same location of the input plane of the JTC. The encrypted image was a single real-valued image. In this paper, the two original images to encrypt are encoded in phase at different locations of the input plane of the JTC and the definitions of the nonlinear operations applied on the JPS differ from the definitions of the nonlinear operations applied in [45]. The security system presented in [46] was performed using a classic DRPE and the Gyrator transform. The two original images to encrypt in [46] represent the real and imaginary parts of a complex-valued image at the input plane of the encryption system and the encrypted image is also a complex-valued image. The double-image encryption in [47] was based on the linear DRPE in the Fresnel domain with the two original images to encrypt encoded in amplitude at the input plane of the encryption system. In [48], an asymmetric double-image encryption system was developed using the linear DRPE in the Fresnel domain with one image encoded in amplitude and the other image encoded in phase placed at the same location of the input plane of the encryption system. The encrypted image generated in [48] was a complex-valued image.

The double-image encryption systems, based on the amplitude encoding for the two images to encrypt and the classical DRPE (linear JTC architectures), are vulnerable to some security attacks [19,30]. In general, the numerical and iterative algorithms for double-image encryption are time consuming and their encryption–decryption process differs from the DRPE technique proposed in [2,5,7,9,20]. We point out that the proposed security system in this work allows for a simultaneous encryption of two images, their decryption, either jointly or separately, and it is a new nonlinear JTC-based encryption system with a high level of security for the single real-valued encrypted image.

## 3. Encryption and Decryption Systems Based on a Nonlinear JTC Architecture in the FD

### 3.1. Encryption System

**Figure 1:**
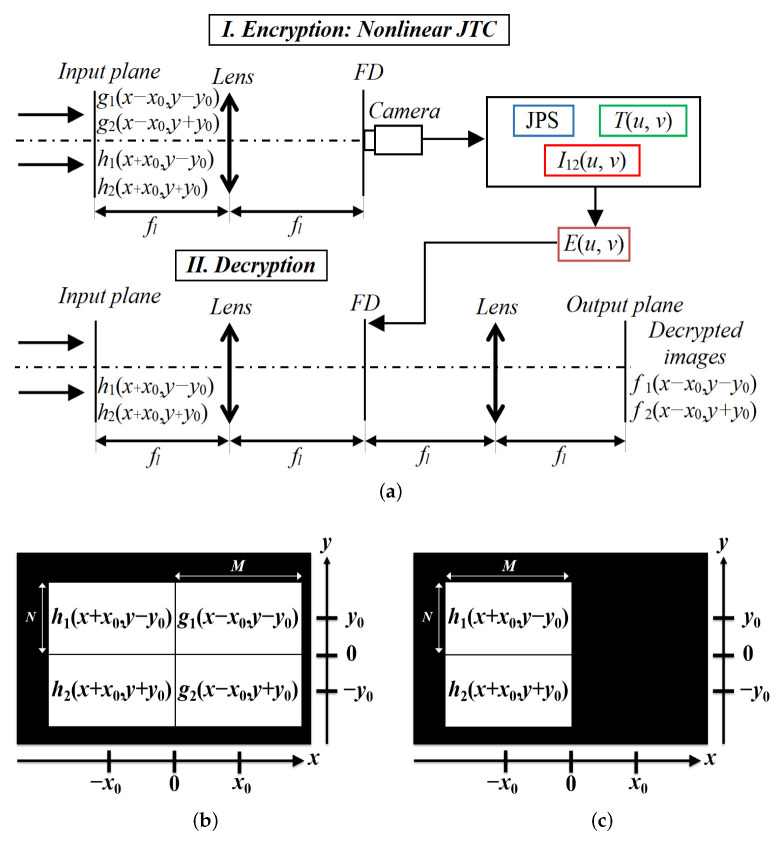
(**a**) The two-image encryption system scheme using a nonlinear JTC architecture (part I) and the decryption system scheme composed of a 4*f*-processor (part II). Data distributions located in the input plane of the: (**b**) encryption system, and (**c**) decryption system.

In this section, we describe the proposed two-image encryption system by using the equations of a nonlinear JTC architecture [2,5,6,7,8,9,10,20,21,22,25,26,28,29]. The two original images to encrypt $f_{1}\left(x,y\right)$ and $f_{2}\left(x,y\right)$ have real values in the interval [0, 1]. These two original images are encoded in phase

(1) $$\begin{array}{c} f_{ph1}\left(x,y\right)=\exp\left\{i2\pi f_{1}\left(x,y\right)\right\},\;f_{ph2}\left(x,y\right)=\exp\left\{i2\pi f_{2}\left(x,y\right)\right\}, \end{array}$$

and the RPMs are defined by the following equation:

(2) $$\begin{array}{c} r_{1}\left(x,y\right)=\exp\left\{i2\pi m_{1}\left(x,y\right)\right\},\;r_{2}\left(x,y\right)=\exp\left\{i2\pi m_{2}\left(x,y\right)\right\},\\ h_{1}\left(x,y\right)=\exp\left\{i2\pi n_{1}\left(x,y\right)\right\},\;h_{2}\left(x,y\right)=\exp\left\{i2\pi n_{2}\left(x,y\right)\right\}, \end{array}$$

where *x* and *y* denote the input coordinates at the spatial domain, $m_{1}\left(x,y\right)$, $m_{2}\left(x,y\right)$, $n_{1}\left(x,y\right)$ and $n_{2}\left(x,y\right)$ are normalized positive function randomly generated, statistically independent and uniformly distributed in the interval [0, 1] [7,9,20]. All the images used in the encryption and decryption systems have M×N pixels size. Figure 1a depicts the encryption system scheme (part I) using a nonlinear JTC architecture and the decryption system scheme (part II) based on a 4*f*-processor.

Figure 1b shows four phase-only data distribution spatially separated for the input plane of the JTC-based encryption system. The first and second data distributions are represented by $g_{1}\left(x,y\right)$ and $g_{2}\left(x,y\right)$, respectively, and these data distributions are defined by the original images to encrypt encoded in phase $f_{ph1}\left(x,y\right)$ and $f_{ph2}\left(x,y\right)$ bonded to the RPMs $r_{1}\left(x,y\right)$ and $r_{2}\left(x,y\right)$, respectively:

(3) $$\begin{array}{c} g_{1}\left(x,y\right)=f_{ph1}\left(x,y\right)r_{1}\left(x,y\right)=\exp\left\{i2\pi \left[f_{1}\left(x,y\right)+m_{1}\left(x,y\right)\right]\right\},\\ g_{2}\left(x,y\right)=f_{ph2}\left(x,y\right)r_{2}\left(x,y\right)=\exp\left\{i2\pi \left[f_{2}\left(x,y\right)+m_{2}\left(x,y\right)\right]\right\}. \end{array}$$

The third and fourth data distributions of the input plane of the JTC are given by the RPMs $h_{1}\left(x,y\right)$ and $h_{2}\left(x,y\right)$, respectively. The data distributions of the input plane of the JTC-based encryption system $g_{1}\left(x,y\right)$, $g_{2}\left(x,y\right)$, $h_{1}\left(x,y\right)$ and $h_{2}\left(x,y\right)$ are located centred at the coordinates $\left(x,y\right)=\left(x_{0},y_{0}\right)$, $\left(x,y\right)=\left(x_{0},-y_{0}\right)$, $\left(x,y\right)=\left(-x_{0},y_{0}\right)$ and $\left(x,y\right)=\left(-x_{0},-y_{0}\right)$, respectively. All four data distributions do not overlap spatially and there is not blank space between them [28].

The JPS is the intensity of the FT (F) of the input plane of the JTC, and it is given by

(4) $$\begin{array}{cc} \mathrm{JPS}(u,v)= & |F\{g_{1}\left(x-x_{0},y-y_{0}\right)+g_{2}\left(x-x_{0},y+y_{0}\right)\\ & +h_{1}\left(x+x_{0},y-y_{0}\right)+h_{2}\left(x+x_{0},y+y_{0}\right)\}{|}^{2}\\ = & |G_{1}\left(u,v\right)e^{-i2\pi (x_{0}u+y_{0}v)}+G_{2}\left(u,v\right)e^{-i2\pi (x_{0}u-y_{0}v)}\\ & +H_{1}\left(u,v\right)e^{-i2\pi (-x_{0}u+y_{0}v)}+H_{2}\left(u,v\right)e^{-i2\pi (-x_{0}u-y_{0}v)}{|}^{2}, \end{array}$$

where *u* and *v* indicate the output coordinates in the FD and the distributions denoted by capital letters represent the FTs of the distributions denoted in lowercase letters.

In the next step of the encryption system, the JPS is modified by two new nonlinear operations with the purpose of obtaining the encrypted image

(5) $$\begin{array}{cc} E(u,v)= & \frac{\mathrm{JPS}\left(u,v\right)-I_{12}\left(u,v\right)}{T(u,v)}\\ = & \frac{1}{T(u,v)}[G_{1}\left(u,v\right)H_{1}^{*}\left(u,v\right)e^{-i2\pi (2x_{0}u)}+G_{1}\left(u,v\right)H_{2}^{*}\left(u,v\right)e^{-i2\pi (2x_{0}u+2y_{0}v)}\\ & G_{2}\left(u,v\right)H_{1}^{*}\left(u,v\right)e^{-i2\pi (2x_{0}u-2y_{0}v)}+G_{2}\left(u,v\right)H_{2}^{*}\left(u,v\right)e^{-i2\pi (2x_{0}u)}\\ & +G_{1}^{*}\left(u,v\right)H_{1}\left(u,v\right)e^{-i2\pi (-2x_{0}u)}+G_{2}^{*}\left(u,v\right)H_{1}\left(u,v\right)e^{-i2\pi (-2x_{0}v+2y_{0}v)}\\ & +G_{1}^{*}\left(u,v\right)H_{2}\left(u,v\right)e^{-i2\pi (-2x_{0}u-2y_{0}v)}+G_{2}^{*}\left(u,v\right)H_{2}\left(u,v\right)e^{-i2\pi (-2x_{0}u)}], \end{array}$$

with

(6) $$\begin{array}{cc} I_{12}\left(u,v\right)= & {\left|F\left\{g_{1}\left(x-x_{0},y-y_{0}\right)+g_{2}\left(x-x_{0},y+y_{0}\right)\right\}\right|}^{2}+T\left(u,v\right),\\ T(u,v)= & {\left|F\left\{h_{1}\left(x+x_{0},y-y_{0}\right)+h_{2}\left(x+x_{0},y+y_{0}\right)\right\}\right|}^{2}. \end{array}$$

The superscript ${}^{*}$ presented in Equation (5) denotes the complex conjugation operation. The two new nonlinear terms given by Equation (6) are introduced in the definition of the encrypted image with the double purpose of improving the quality of the decrypted images and increasing the security of the encrypted image against several plaintext attacks, in a similar way as it was proposed in [5,20,21,25,28,44,49]. The new nonlinear term represented by $I_{12}\left(u,v\right)$ has cross-correlation terms in the FD between $G_{1}\left(u,v\right)$ and $G_{2}\left(u,v\right)$, and also between $H_{1}\left(u,v\right)$ and $H_{2}\left(u,v\right)$, besides the usual terms given by the following intensity distributions ${\left|G_{1}\left(u,v\right)\right|}^{2}$, ${\left|G_{2}\left(u,v\right)\right|}^{2}$, ${\left|H_{1}\left(u,v\right)\right|}^{2}$ and ${\left|H_{2}\left(u,v\right)\right|}^{2}$ used in [5,20,44]. The subtraction of $I_{12}\left(u,v\right)$ from the JPS(u,v) allows a correct implementation of the DRPE technique because all the terms presented in the numerator of Equation (5) are cross-correlation terms in the FD between $G_{i}\left(u,v\right)$ and $H_{j}\left(u,v\right)$ with i=1,2 and j=1,2. The nonlinear term T(u,v) introduced in Equation (5) allows the proposed security system to more closely approach the output result to the original DRPE technique [5,7,20]. Since the JPS modifications consist of nonlinear operations, they also contribute to increasing the overall security of the processor against plaintext attacks in comparison with the other linear systems.

The encrypted image E(u,v) given by Equation (5) is computed from three intensities distributions, which can be sequentially captured when different data distributions are separately displayed at the input plane of the JTC. Therefore, the encrypted image is a real-valued distribution and the security keys of the encryption system are given by the four RPMs ($r_{1}\left(x,y\right)$, $r_{2}\left(x,y\right)$, $h_{1}\left(x,y\right)$ and $h_{2}\left(x,y\right)$). The proposed encryption and decryption systems can be implemented by using optical FT setups and the three intensities distributions needed to compute the encrypted image can be performed at the speed of light [6,8]. A 2*f* and 4*f* optical processors can be used in order to implement the proposed encryption and decryption systems, respectively. The two nonlinear operations applied to the JPS in Equation (5) are performed by digital computation. The numerical computational complexity of the encryption system is mainly given by the three two-dimensional (2D) fast Fourier transforms (FFTs) to compute the terms JPS(u,v), $I_{12}\left(u,v\right)$ and T(u,v) utilized in the definition of the encrypted image. The term JPS(u,v) is computed by applying the 2D FFT to an image that has 2M×2N pixels size and the terms $I_{12}\left(u,v\right)$ and T(u,v) are computed by applying the 2D FFT to two different images that have M×2N pixels size.

### 3.2. Decryption System

The decryption system scheme is presented in Figure 1a (part II), and it is based on two successive FTs (4*f*-processor). In the first step of the decryption system, the third and fourth data distributions $h_{1}\left(x,y\right)$ and $h_{2}\left(x,y\right)$ are placed at the input plane of the decryption system (Figure 1c) at coordinates $\left(x,y\right)=\left(-x_{0},y_{0}\right)$ and $\left(x,y\right)=\left(-x_{0},-y_{0}\right)$, respectively, and this input plane is Fourier transformed; the result of this transformation is multiplied by the encrypted image E(u,v) to obtain

(7) $$\begin{array}{cc} D(u,v)= & E\left(u,v\right)F\left\{h_{1}\left(x+x_{0},y-y_{0}\right)+h_{2}\left(x+x_{0},y+y_{0}\right)\right\}\\ = & E\left(u,v\right)\left[H_{1}\left(u,v\right)e^{-i2\pi (-x_{0}u+y_{0}v)}+H_{2}\left(u,v\right)e^{-i2\pi (-x_{0}u-y_{0}v)}\right]\\ = & \frac{1}{T(u,v)}[G_{1}\left(u,v\right){\left|H_{1}\left(u,v\right)\right|}^{2}e^{-i2\pi (x_{0}u+y_{0}v)}+G_{1}\left(u,v\right){\left|H_{2}\left(u,v\right)\right|}^{2}e^{-i2\pi (x_{0}u+y_{0}v)}\\ & +G_{1}\left(u,v\right)H_{1}^{*}\left(u,v\right)H_{2}\left(u,v\right)e^{-i2\pi (x_{0}u-y_{0}v)}+G_{1}\left(u,v\right)H_{1}\left(u,v\right)H_{2}^{*}\left(u,v\right)e^{-i2\pi (x_{0}u+3y_{0}v)}\\ & +G_{2}\left(u,v\right){\left|H_{1}\left(u,v\right)\right|}^{2}e^{-i2\pi (x_{0}u-y_{0}v)}+G_{2}\left(u,v\right){\left|H_{2}\left(u,v\right)\right|}^{2}e^{-i2\pi (x_{0}u-y_{0}v)}\\ & +G_{2}\left(u,v\right)H_{1}^{*}\left(u,v\right)H_{2}\left(u,v\right)e^{-i2\pi (x_{0}u-3y_{0}v)}+G_{2}\left(u,v\right)H_{1}\left(u,v\right)H_{2}^{*}\left(u,v\right)e^{-i2\pi (x_{0}u+y_{0}v)}\\ & +G_{1}^{*}\left(u,v\right)H_{1}^{2}\left(u,v\right)e^{-i2\pi (-3x_{0}u+y_{0}v)}+G_{1}^{*}\left(u,v\right)H_{1}\left(u,v\right)H_{2}\left(u,v\right)e^{-i2\pi (-3x_{0}u-y_{0}v)}\\ & +G_{2}^{*}\left(u,v\right)H_{1}^{2}\left(u,v\right)e^{-i2\pi (-3x_{0}u+3y_{0}v)}+G_{2}^{*}\left(u,v\right)H_{1}\left(u,v\right)H_{2}\left(u,v\right)e^{-i2\pi (-3x_{0}u+y_{0}v)}\\ & +G_{1}^{*}\left(u,v\right)H_{1}\left(u,v\right)H_{2}\left(u,v\right)e^{-i2\pi (-3x_{0}u-y_{0}v)}+G_{1}^{*}\left(u,v\right)H_{2}^{2}\left(u,v\right)e^{-i2\pi (-3x_{0}u-3y_{0}v)}\\ & +G_{2}^{*}\left(u,v\right)H_{1}\left(u,v\right)H_{2}\left(u,v\right)e^{-i2\pi (-3x_{0}u+y_{0}v)}+G_{2}^{*}\left(u,v\right)H_{2}^{2}\left(u,v\right)e^{-i2\pi (-3x_{0}u-y_{0}v)}]. \end{array}$$

The output plane of the decryption system is given by the inverse FT of Equation (7). This output plane has several data distributions spatially separated. The first eight terms of Equation (7) are the most interesting terms since they retain the two original images to be decrypted. The first four terms of Equation (7) allow for recovering the data distribution $g_{1}\left(x,y\right)$, centred at coordinates $\left(x,y\right)=\left(x_{0},y_{0}\right)$ and the data distribution $g_{2}\left(x,y\right)$ placed at coordinates $\left(x,y\right)=\left(x_{0},-y_{0}\right)$ is retrieved by using the fourth to the eighth term of the same equation. The last eight terms of Equation (7) are noisy data distributions at the output plane of the decryption system, and these terms are spatially separated from the sought distributions $g_{1}\left(x,y\right)$ and $g_{2}\left(x,y\right)$. The retrieval of the data distribution $g_{1}\left(x,y\right)$ and $g_{2}\left(x,y\right)$ from Equation (7) is given by

(8) $$\begin{array}{cc} d_{1-4}\left(x,y\right)= & F^{-1}\{\frac{G_{1}\left(u,v\right)e^{-i2\pi (x_{0}u+y_{0}v)}}{T(u,v)}[{\left|H_{1}\left(u,v\right)\right|}^{2}+{\left|H_{2}\left(u,v\right)\right|}^{2}\\ & \;+H_{1}^{*}\left(u,v\right)H_{2}\left(u,v\right)e^{-i2\pi (-2y_{0}v)}+H_{1}\left(u,v\right)H_{2}^{*}\left(u,v\right)e^{-i2\pi (2y_{0}v)}]\}\\ = & g_{1}\left(x-x_{0},y-y_{0}\right),\\ d_{5-8}\left(x,y\right)= & F^{-1}\{\frac{G_{2}\left(u,v\right)e^{-i2\pi (x_{0}u-y_{0}v)}}{T(u,v)}[{\left|H_{1}\left(u,v\right)\right|}^{2}+{\left|H_{2}\left(u,v\right)\right|}^{2}\\ & \;+H_{1}^{*}\left(u,v\right)H_{2}\left(u,v\right)e^{-i2\pi (-2y_{0}v)}+H_{1}\left(u,v\right)H_{2}^{*}\left(u,v\right)e^{-i2\pi (2y_{0}v)}]\}\\ = & g_{2}\left(x-x_{0},y+y_{0}\right). \end{array}$$

Finally, the decrypted images are obtained from the previous equation as follows:

(9) $$\begin{array}{c} 2\pi {\hat{f}}_{1}\left(x-x_{0},y-y_{0}\right)=\arg\left\{d_{1-4}\left(x,y\right)r_{1}^{*}\left(x-x_{0},y-y_{0}\right)\right\},\\ 2\pi {\hat{f}}_{2}\left(x-x_{0},y+y_{0}\right)=\arg\left\{d_{5-8}\left(x,y\right)r_{2}^{*}\left(x-x_{0},y+y_{0}\right)\right\}, \end{array}$$

where arg denotes the phase of a complex-valued function. In order to obtain the decrypted images ${\hat{f}}_{1}\left(x,y\right)$ and ${\hat{f}}_{2}\left(x,y\right)$ as replicas of the original images $f_{1}\left(x,y\right)$ and $f_{2}\left(x,y\right)$, respectively, the four security keys given by the RPMs $r_{1}\left(x,y\right)$, $r_{2}\left(x,y\right)$, $h_{1}\left(x,y\right)$ and $h_{2}\left(x,y\right)$ used in the decryption system have to be the same as the security keys used in the encryption system. We remark that the new nonlinear operations introduced in the JPS given by the terms $I_{12}\left(u,v\right)$ and T(u,v), allow for the retrieval of the two original images in Equation (9). The proposed decryption system can be implemented by using two optical FTs which can be performed at the speed of light [6,8]. The numerical computational complexity of the decryption system is mainly given by the computation of two successive 2D FFTs applied to two different images that have 2M×2N pixels size. These two sequentially 2D FFTs are needed to obtain the output plane of the decryption system.

## 4. Simulation Results

**Figure 2:**
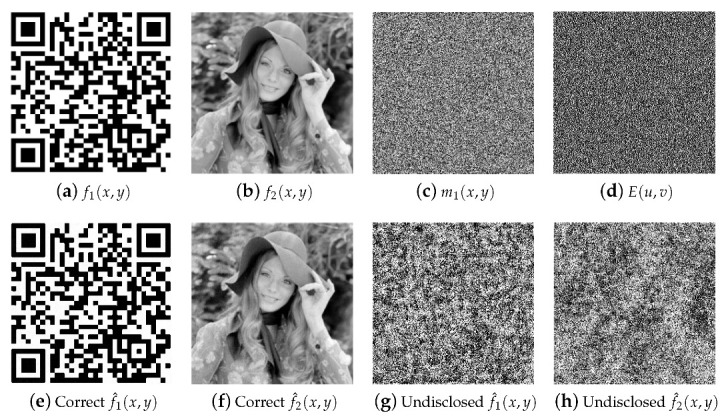
(**a**,**b**) Original images to be encrypted $f_{1}\left(x,y\right)$ and $f_{2}\left(x,y\right)$, respectively; (**c**) image of the random distribution $m_{1}\left(x,y\right)$ of the RPM $r_{1}\left(x,y\right)$; (**d**) encrypted image E(u,v); (**e**,**f**) correct decrypted images ${\hat{f}}_{1}\left(x,y\right)$ and ${\hat{f}}_{2}\left(x,y\right)$, respectively, using the right four security RPMs; (**g**,**h**) wrong decrypted images ${\hat{f}}_{1}\left(x,y\right)$ and ${\hat{f}}_{2}\left(x,y\right)$, respectively, when the incorrect security key RPM $h_{1}\left(x,y\right)$ and the other three correct security keys RPMs are used in the decryption system.

The simulation results for the encryption and decryption systems described in Section 3 are depicted in Figure 2. All the images utilized in this simulation test have a resolution of 256×256 pixels (M=N=256). The original images to be encrypted $f_{1}\left(x,y\right)$ and $f_{2}\left(x,y\right)$ are displayed in Figure 2a and Figure 2b, respectively. Function $f_{1}\left(x,y\right)$ is a binary image with real values of 0 or 1. Function $f_{2}\left(x,y\right)$ is a grayscale image with real values in the interval [0, 1]. The grayscale image of the random distribution $m_{1}\left(x,y\right)$ of the RPM $r_{1}\left(x,y\right)$ is shown in Figure 2c. The grayscale images of the random distributions $m_{2}\left(x,y\right)$, $n_{1}\left(x,y\right)$ and $n_{2}\left(x,y\right)$ of the RPMs $r_{2}\left(x,y\right)$, $h_{1}\left(x,y\right)$ and $h_{2}\left(x,y\right)$, respectively, have different values but similar appearance to the image depicted in Figure 2c.

The image depicted in Figure 2d corresponds to the real-valued encrypted image E(u,v) with a noisy appearance which does not reveal any information about any of the original images $f_{1}\left(x,y\right)$ and $f_{2}\left(x,y\right)$. Figure 2e,f show the decrypted images ${\hat{f}}_{1}\left(x,y\right)$ and ${\hat{f}}_{2}\left(x,y\right)$ using the same values of the security keys (the four RPMs) that were used in the encryption system. These two decrypted images have been obtained through the whole process represented by Equations (7)–(9) and depict the magnified region centred at coordinates $\left(x,y\right)=\left(x_{0},y_{0}\right)$ and $\left(x,y\right)=\left(x_{0},-y_{0}\right)$ of the output plane of the decryption system. The decrypted images presented in Figure 2g,h correspond to the use of a wrong security key RPM $h_{1}\left(x,y\right)$ along with the other three correct security keys RPMs used in the decryption stage. If the other security keys’ RPMs $r_{1}\left(x,y\right)$, $r_{2}\left(x,y\right)$ or $h_{2}\left(x,y\right)$ are wrong in the decryption system, the decrypted images will be noisy distributions very similar to the images presented in Figure 2g,h. Therefore, all correct security key RPMs are required in the decryption system in order to obtain a meaningful simultaneous retrieval of the two original images.

The root mean square error (RMSE) is used in order to evaluate the quality of the decrypted images, and this RMSE is given by [20]

(10) $$\mathrm{RMSE}={\left(\frac{\sum_{x=1}^{M}\sum_{y=1}^{N}{\left[f\left(x,y\right)-\hat{f}\left(x,y\right)\right]}^{2}}{\sum_{x=1}^{M}\sum_{y=1}^{N}{\left[f\left(x,y\right)\right]}^{2}}\right)}^{\frac{1}{2}}.$$

The values of the RMSE are in the interval of [0,1]. The best quality of the decrypted images would be for RMSE values close or equal to 0. Bad quality decrypted images will obtain RMSE values close to 1. The RMSEs between the original images of Figure 2a,b and the correct decrypted images of Figure 2e,f are 0.0375 and 0.0279, respectively. The RMSEs for the case of the wrong decrpyted images of Figure 2g,h with respect to the original images of Figure 2a,b are 0.91 and 0.82, respectively.

There exists the possibility to separately retrieve the correct decrypted image ${\hat{f}}_{1}\left(x,y\right)$ or ${\hat{f}}_{2}\left(x,y\right)$ under certain combinations for the values of the security keys, this fact is due to the results obtained in Equation (9). Thus, when the security keys $r_{1}\left(x,y\right)$, $h_{1}\left(x,y\right)$ and $h_{2}\left(x,y\right)$ are correct and $r_{2}\left(x,y\right)$ is wrong in the decryption system, we obtain the same correct decrypted image ${\hat{f}}_{1}\left(x,y\right)$ depicted in Figure 2e and a noisy decrypted image ${\hat{f}}_{2}\left(x,y\right)$ very similar to the image shown in Figure 2h. The other case uses the correct security keys $r_{2}\left(x,y\right)$, $h_{1}\left(x,y\right)$ and $h_{2}\left(x,y\right)$, and the wrong security key $r_{1}\left(x,y\right)$ in the decryption system, with the purpose of recovering the same correct decrypted image ${\hat{f}}_{2}\left(x,y\right)$ presented in Figure 2f and a noisy decrypted image ${\hat{f}}_{1}\left(x,y\right)$ very similar to the image displayed in Figure 2g.

### Key Space and Robustness to Attacks

The key space of the encryption–decryption system of this work is given by all of the possible combinations of the four security keys RPMs ($r_{1}\left(x,y\right)$, $r_{2}\left(x,y\right)$, $h_{1}\left(x,y\right)$ and $h_{2}\left(x,y\right)$). The RPMs have a random distribution, which is a grayscale image with 256×256 pixels size and every pixel of this image has 256 different values. Therefore, the all possible combinations required to retrieve the four RPMs is of the order of $256^{4(256)(256)}=256^{262144}$. For this reason, a brute force attack applied to the proposed encryption–decryption system is impractical due to the larger key space of this security system [50].

Several JTC architectures utilized in security systems have been shown to be vulnerable to several attacks, such as the CPA [15], the KPA [16,17] and the COA [18], among others, due to their linearity. The nonlinear JTC architectures presented in references [5,20,21,25,28,44] improved the security for the JTC-based encryption system against CPA, KPA and COA. The phase encoding of the input plane of the proposed JTC-based encryption system is a nonlinear operation that allows for an improved security against COA described in [18] because this COA is based on an iterative phase retrieval algorithm that permits retrieving a single plaintext (original image to be encrypted) encoded in amplitude by using only a ciphertext (encrypted image). In the current proposal, the two primary images or plaintexts are phase-encoded at the JTC input plane. Thus, this COA will hardly retrieve the correct values for the two phase-encoded plaintexts, and it also cannot retrieve the four security keys RPMs ($r_{1}\left(x,y\right)$, $r_{2}\left(x,y\right)$, $h_{1}\left(x,y\right)$ and $h_{2}\left(x,y\right)$) [5,18,28,30,44]. The two new nonlinear operations applied to the JPS to compute the encrypted image are very important in order to break the linearity of the JTC-based encryption systems and to increase the security of the encrypted image against CPA and KPA, as it was proved in [5,20,21,28]. The introduction of this two new nonlinear operations over the JPS allows the simultaneous encryption of the two phase-encoded original images (plaintexts) by implementing a DRPE technique with four security keys’ RPMs. The CPA and KPA described in references [15] and [16,17], respectively, were specifically designed to find only one security key RPM (h(x,y)) because there was a single plaintext encoded in amplitude for the linear JTC-based encryption system under attack. These CPA and KPA would not be able to find the other RPM (r(x,y)). Therefore, these CPA and KPA would probably fail if they were applied to the current nonlinear system proposed in this work because these attacks would not be able to retrieve the correct values of the four security keys’ RPMs.

Recently, the DRPE has been shown to be vulnerable to an attack implemented with deep learning [19]. Such an attack was designed for a linear 4*f*-processor system whose single image to encrypt was encoded in amplitude (real-valued distribution). Therefore, the attack described in reference [19] is not suitable for the security system of this paper, and it is not able to retrieve the four security keys RPMs because the proposed encryption system in this work is designed with a nonlinear 2*f*-processor system (JTC architecture), and the two original images to encrypt are encoded in phase (complex-valued distribution).

## 5. Conclusions

In this paper, we have presented a novel extension of the single image encryption system based on a nonlinear JTC to a double image encryption system using new nonlinear modifications applied to the JTC architecture in the FD. The original images to be encrypted are two images (either binary or grayscale) with or without relationship between them. The security keys of the proposed encryption–decryption system are given by four RPMs. The new nonlinear operations applied on the JPS in order to obtain the encrypted image have allowed for retrieving the two correct decrypted images and an improvement in the security of the proposed encryption–decryption system against brute force and plaintext attacks due to a correct implementation of the DRPE technique based on a nonlinear JTC architecture. The right simultaneous retrieval of the two original images at the output plane of the decryption system is only possible when the same four security keys RPMs of the encryption system are applied in the decryption system. Decryption of the original images with the best image quality is only achieved by using the correct four security keys RPMs and the new nonlinear operations applied on the JPS. The design of the proposed security system has allowed the possibility to separately retrieve one of the two correct decrypted images. The proposed encryption and decryption systems in this work can be implemented using optical setups based on 2*f* and 4*f* processors, respectively, except for the nonlinear operations applied to the JPS which have to be digitally computed. Finally, the larger key space, the nonlinear modifications in the JTC architecture and the phase encoding of the two original images to encrypt allowed an improved security for the encrypted image against several attacks because the linearity of the previous JTC-based encryption systems was broken and the design of the proposed nonlinear security system caused the simultaneous encryption of the two original images by implementing a correct DRPE technique with four security keys RPMs.

## Data Availability

The supporting information can be found from the corresponding author.

## Abbreviations

The following abbreviations are used in this manuscript:

2D	Two-Dimensional
CPA	Chosen-Plaintext Attack
COA	Ciphertext-Only Attack
DRPE	Double Random Phase Encoding
FFT	Fast Fourier Transform
FD	Fourier Domain
FT	Fourier Transform
KPA	Known-Plaintext Attack
JTC	Joint Transform Correlator
JPS	Joint Power Spectrum
RPMs	Random Phase Masks
RMSE	Root Mean Square Error
